# Supplementary material for: Molecular Dynamics Simulation of Polyacrylamide Adsorption on Cellulose Nanocrystals
Source: Nanomaterials (Basel). 2020 Jun 28;10(7):1256. doi: 10.3390/nano10071256 (PMC7408107; doi:10.3390/nano10071256)
Supplement: Supplementary file 1 [file nanomaterials-10-01256-s001.pdf]

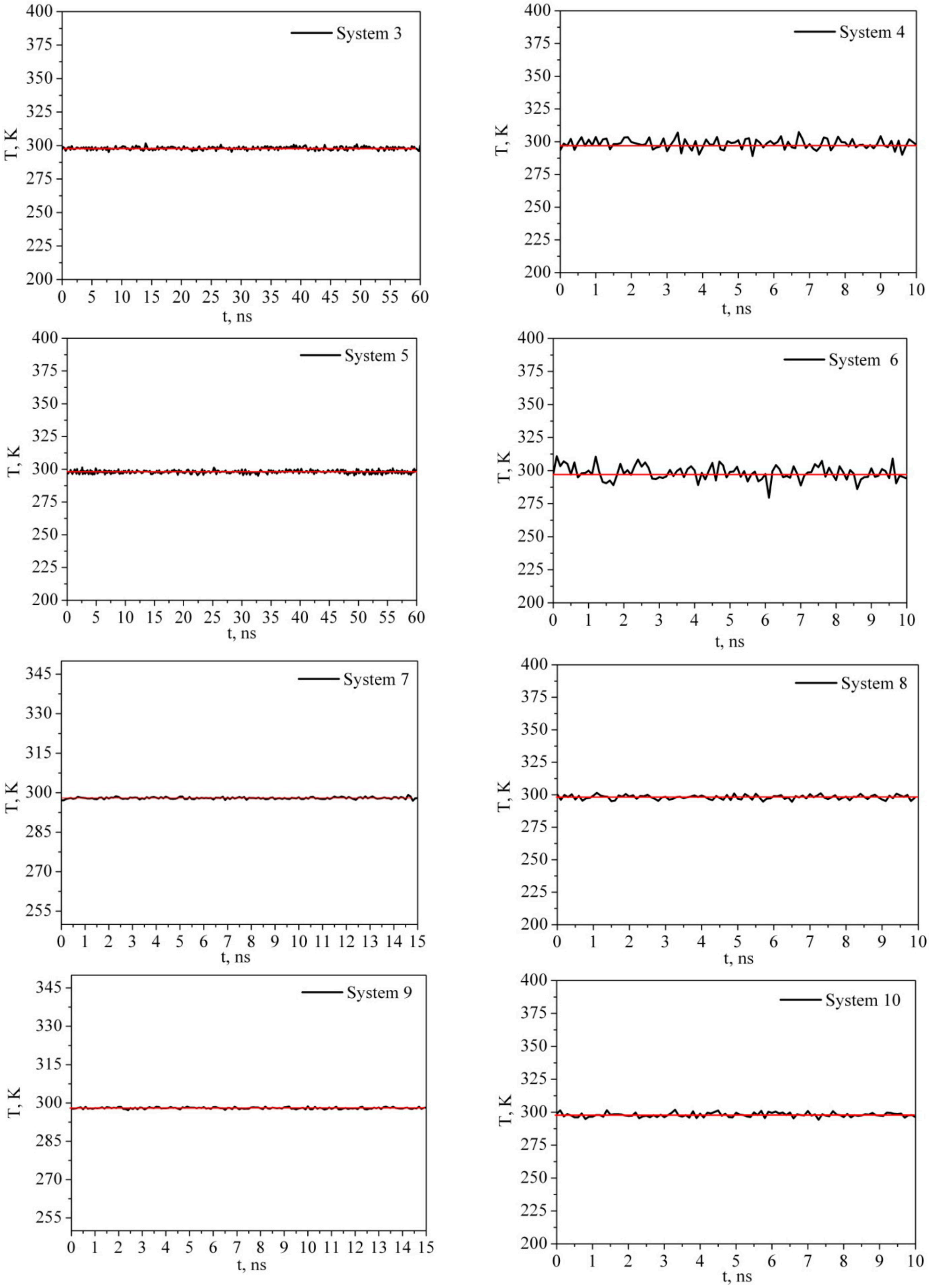

Figure S1. Time dependence of the system temperature

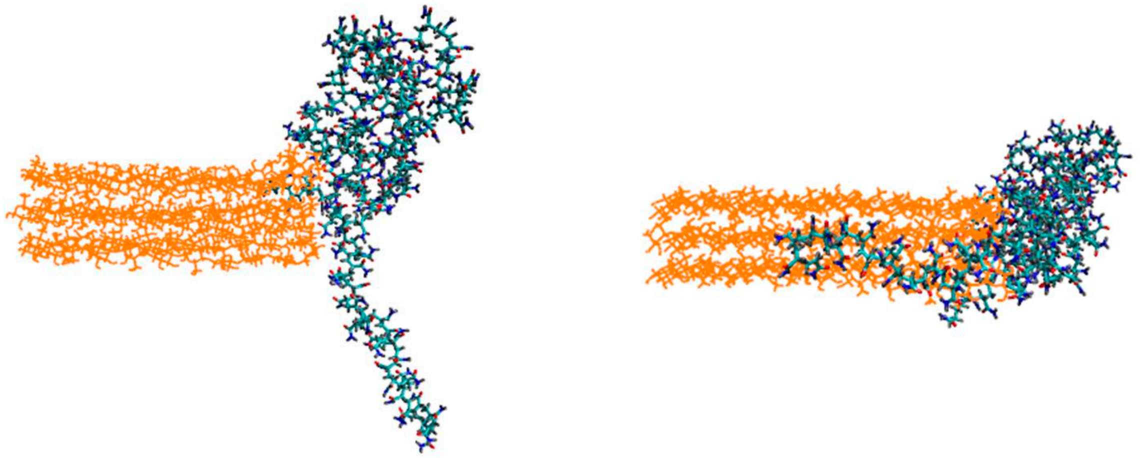

(**a**) (**b**)

Figure S2. The instant snapshots of System 6 at 7 ns (**a**) and 7.8 ns (**b**). These snapshots explain the jump in $N_C(t)$ at ≈7.5 ns.
